# The stroke transitional care intervention for older adults with stroke and multimorbidity: a multisite pragmatic randomized controlled trial

**DOI:** 10.1186/s12877-023-04403-1

**Published:** 2023-10-24

**Authors:** Maureen Markle-Reid, Kathryn Fisher, Kimberly M. Walker, Marla Beauchamp, Jill I. Cameron, David Dayler, Rebecca Fleck, Amiram Gafni, Rebecca Ganann, Ken Hajas, Barbara Koetsier, Robert Mahony, Chris Pollard, Jim Prescott, Tammy Rooke, Carly Whitmore

**Affiliations:** 1 School of Nursing, Faculty of Health Sciences, McMaster University, 1280 Main Street West, Room HSc3N25, Hamilton, ON L8S 4K1 Canada; 2 Health Research Methods, Department of Health, Evidence and Impact, Faculty of Health Sciences, and the Centre of Health Economics and Policy Analysis, McMaster University, 1280 Main Street West, HSC 2C, Hamilton, ON L8S 4K1 Canada; 3 Aging, Community and Health Research Unit, School of Nursing, Faculty of Health Sciences, McMaster University, 1280 Main Street West, Room HSc3N25, Hamilton, ON L8S 4K1 Canada; 4 McMaster Institute for Research On Aging, McMaster University, Hamilton, ON Canada; 5 Upstream Lab, MAP Centre for Urban Health Solutions, St. Michael’s Hospital, 209 Victoria Street, Ontario M5B 1T8 Toronto, Canada; 6 Department of Occupational Science and Occupational Therapy, Rehabilitation Sciences Institute, Temerty Faculty of Medicine, University of Toronto, 160-500 University Ave, Toronto, ON M5V 1V7 Canada; 7 Rehabilitation Program, Parkwood Institute, St. Joseph’s Health Care London, 268 Grosvenor Street, Ontario N6A 4V2 London, Canada; 8 Hotel Dieu Shaver Health, and Rehabilitation Centre, 541 Glenridge Ave, St. Catherines, ON L2T 4C2 Canada; 9 CarePartners, 139 Washburn Drive, Kitchener, ON N2R 1S1 Canada

**Keywords:** Older adults, Multimorbidity, Stroke rehabilitation, Transitions, Community-based care, Healthcare intervention, Effectiveness, Costs

## Abstract

**Background:**

This study aimed to test, in real-world clinical practice, the effectiveness of a Transitional Care Stroke Intervention (TCSI) compared to usual care on health outcomes, self-management, patient experience, and health and social service use costs in older adults (≥ 55 years) with stroke and multimorbidity (≥ 2 chronic conditions).

**Methods:**

This pragmatic randomized controlled trial (RCT) included older adults discharged from hospital to community with stroke and multimorbidity using outpatient stroke rehabilitation services in two communities in Ontario, Canada. Participants were randomized 1:1 to usual care (control group) or usual care plus the 6-month TCSI (intervention group). The TCSI was delivered virtually by an interprofessional (IP) team, and included care coordination/system navigation support, phone/video visits, monthly IP team conferences, and an online resource to support system navigation. The primary outcome was risk of hospital readmission (all cause) after six-months. Secondary outcomes included physical and mental functioning, stroke self-management, patient experience, and health and social service use costs. The intention-to-treat principle was used to conduct the primary and secondary analyses.

**Results:**

Ninety participants were enrolled (44 intervention, 46 control); 11 (12%) participants were lost to follow-up, leaving 79 (39 intervention, 40 control). No significant between-group differences were seen for baseline to six-month risk of hospital readmission. Differences favouring the intervention group were seen in the following secondary outcomes: physical functioning (SF-12 PCS mean difference: 5.10; 95% CI: 1.58–8.62, *p* = 0.005), stroke self-management (Southampton Stroke Self-Management Questionnaire mean difference: 6.00; 95% CI: 0.51—11.50, *p* = 0.03), and patient experience (Person-Centred Coordinated Care Experiences Questionnaire mean difference: 2.64, 95% CI: 0.81, 4.47, *p* = 0.005). No between-group differences were found in total healthcare costs or other secondary outcomes.

**Conclusions:**

Although participation in the TCSI did not impact hospital readmissions, there were improvements in physical functioning, stroke self-management and patient experience in older adults with stroke and multimorbidity without increasing total healthcare costs. Challenges associated with the COVID-19 pandemic, including the shift from in-person to virtual delivery, and re-deployment of interventionists could have influenced the results. A larger pragmatic RCT is needed to determine intervention effectiveness in diverse geographic settings and ethno-cultural populations and examine intervention scalability.

**Trial registration:**

ClinicalTrials.gov Identifier: NCT04278794. Registered May 2, 2020.

## Introduction

Stroke-related mortality in Canada during the 10-year period 2003–2013 decreased by 26.1% (38.0 to 28.1 per 1,000), consistent with the decline in stroke incidence of 21.6% which is observed from 2003–2011 in Ontario, Canada’s most populated province [1, 2]. A slight decline in stroke incidence has also been reported globally for high-income countries [3]. Raised awareness and better management of risk factors have undoubtedly contributed to this reduced incidence [1]. The development and delivery of reperfusion-based treatments have led to increasing survival rates following acute stroke [4], as has the reconfiguration of acute stroke care services focused on providing rapid access to multidisciplinary stroke teams with state-of-the-art diagnostic and monitoring equipment [5]. In Ontario (Canada), stroke patients’ access to hyperacute care has grown in the past few years, largely due to steady growth in access to endovascular therapy [6]. The UK has undergone one of the world’s largest transformations having initially established eight hyper acute stroke units (HASUs) in Central London in 2008 that were subsequently rolled out across the UK [7–10]. HASU’s aim to provide rapid assessment and diagnosis; around-the-clock access to stroke specialists; immediate MRIs and scans; and 24-h physiological and neurological monitoring, treatment, and early rehabilitation in the first two to three days following stroke, after which patients are discharged to a local acute stroke unit, rehabilitation unit, or home [11].

Mobile Stroke Units (MSUs) are rapidly expanding on a global scale, having been implemented in 23 locations in the US as well as countries such as Germany, Australia, Norway, India, Argentina, Thailand, and Canada. MSUs provide rapid access to diagnostic and treatment services via a specialized ambulance equipped with CT scanner, stroke-specific medications, point-of-care laboratory testing, and other supplies and capabilities needed to treat ischemic stroke patients.

Australia’s national system of clinical audits to ensure that evidence-based acute stroke care aligns with best practices has resulted in a range of improvements over the period 1999 to 2019 in areas including rapid assessment/management for patients with transient ischemic attack, access to stroke units, receipt of thrombolysis services, risk factor advice, and carer training [12]. Other countries conduct similar audits, including the UK, Sweden, Germany, the Republic of Ireland, the Netherlands, Norway and Finland. These audits continue to shape the provision and structure of stroke care, including the organization of ESD, community stroke services, and coordination of HASU services [13].

Alongside these improvements in the organization and provision of acute stroke care services has been relatively short hospital length of stays in most developed countries, thus most of the stroke patients’ recovery occurs after the patient is discharged from the hospital [14]. Up to 60% of older adults hospitalized with a stroke are discharged directly home after an acute care or in-patient rehabilitation hospital stay, and up to 60% will require ongoing rehabilitation in the community that supports continued recovery and re-integration to life after stroke [15]. This trend toward shorter hospital lengths of stay and increasing numbers of stroke survivors’ being discharged to the community, underscores the importance of post-acute systems of care such as early supported discharge (ESD) and transitional care (TC) services. ESD services consist of a multidisciplinary specialized team that provides rehabilitation in a home or community-based setting for selected patients at the same intensity and mix of skills as an acute stroke unit and can include a home visit prior to discharge to tailor the care plan to the patient’s environment [11, 13]. ESD can include a wide range of services from full service with a mobile rehabilitation team through to minimal counselling prior to discharge [16]. Systematic reviews have shown that ESD can reduce disability, length of time in hospital, and healthcare costs for selected people with stroke [16, 17]. Unfortunately, most of the trials included in these reviews are at least 20 years old and occurred prior to the modern reconfigurations of acute stroke services, thus recent research is needed to determine establish/verify these effects.

TC includes a broad spectrum of services that are often included in the same category as ESD for post-acute care of stroke [18]. Transitions are defined as movements of patients across healthcare settings, locations, and providers, and are “marked by changes in patients’ physical, mental, emotional, and cognitive capacities” [19] (p. 790). TC includes the development of individualized discharge care plans as well as a range of support services that assist patients with managing changes – e.g., providing comfort, listening to problems, providing self-management training, and screening/assessing health status and the home environment to assist with emotional and mental health challenges and address accessibility and safety concerns; providing education and instruction on exercises and the use of associated rehabilitation devices to address physical and cognitive challenges; providing training, support with household chores, and facilitating linkages to community-based resources to assist with instrumental activities etc. [19].

The existing evidence of the effectiveness of TC services for stroke patients is mixed. Jee et al.’s [18] recent review of TC and ESD services did not find large effect sizes, reporting only tendencies for decreasing hospital length of stay and better activities of daily living (ADL) and no differences in patient or caregiver outcomes. Duncan et al.’s [20] study of the effectiveness of a nationwide TC stroke intervention in the US was uncertain. Wang et al. [21] found limited evidence for the effect of TC interventions on mortality and some evidence of improvement in ADLs, with the best evidence of effect coming from home-visiting programs involving multidisciplinary teams. Reviews of TC models more generally (across various diseases) found that those involving more components, particularly those focused on fostering education/learning and self-management, were more likely to result in successful transitions [19]. On balance, uncertainties remain regarding the extent to which TC interventions can prevent adverse outcomes, which components are most effective and for which outcomes, and which populations are most likely to benefit [22].

ESD and TC services typically last a maximum of 3 months, after which patients requiring further rehabilitation are transferred to other community services [11]. Community-based care programs typically offer services located in the community, in the patient’s home, or in group living situations. These services may include home care, although in Canada home care services for stroke patients are normally limited to patients with more severe stroke or those that are unable to access community-based programs (e.g., people that do not have an informal carer, people with physical limitations that preclude safe travel). Community-based programs include many of the same services delivered by ESD and TC programs (e.g., tailored rehabilitation exercises, education, self-management training) and reviews have linked these programs to improvements in physical functioning and ADLs; however, more research on long-term effects is needed, with the limited evidence suggesting these programs may be less effective for disease-related conditions and quality of life [3, 23].

Nevertheless, there have been continued efforts to develop and implement clinical guidelines on stroke rehabilitation that emphasize services to support transitions as patients move from one stage to another in their stroke recovery. One example is Part Two of the Canadian Stroke Best Practice Recommendations, which focuses entirely on transitions with recommendations on: 1) *supporting stroke patients & their families* (e.g., screening & assessment, use of telemedicine, information on peer support), 2) *education* (e.g., individualized assessment of needs & development of education plan, self-management skills training), 3) *interprofessional care planning* (e.g., deliberate plan for collaboration and information transfer among health care providers, goal-setting with patient/family/caregiver, home assessments), 4) *community participation* (e.g., education and screening, exercise programs, rehabilitation and management to return to driving, referrals to vocational services to resume social and life roles, advance care and palliative care), and 5) *long-term care* (e.g., assessment, rehabilitation and restorative care) [19].

Ultimately, the burden of stroke in Canada is expected to rise due to an increase in the number of people experiencing and/or surviving a stroke, which is a consequence of various forces including population growth, aging, and improvements in acute stroke care [1]. Similar trends are expected in countries across the globe, with stroke-related disability burden showing a 12% increase worldwide since 1990 [24]. This “underscores a great need for strengthening stroke rehabilitation systems as a mainstay of treating stroke-related illness.” [24] (pg. 403). As noted above, the evidence of the long-term effectiveness of community-based rehabilitations services is lacking. However, we do know that the significant accomplishments seen in acute stroke care have not been matched by improvements to post-acute stroke rehabilitation services in the community. In Ontario (Canada), many stroke patients do not receive the required intensity of rehabilitation therapy recommended by the Canadian Stroke Best Practices (received 69 min/day compared to the recommended 180 min/day), and only 50% of stroke patients access post-acute (inpatient and/or community-based) rehabilitation [6].

Studies from other countries similarly report that stroke survivors are unable to access essential rehabilitation services following hospital discharge [25–27]. A meta-ethnographic systematic review attributed lack of community services and primary care support to the feelings of abandonment that stroke survivors and their caregivers report following hospital discharge [26, 28]. This review included studies from diverse healthcare contexts (e.g., North America, UK, Australia, Northern Europe, Iran) and settings (e.g., municipal, rural, ethnic minorities). The observed lack of community-based services is likely to be even more acute in low- and middle-income countries, with higher population densities and fewer patients able to access and/or pay for services – e.g., Zeng et al. [3] that as few as 40% of patients with stroke are able to afford services compared to 90% + in high-income countries. This suggests that access is a global concern, that can be expected to be more significant in countries with a large geographic range and/or proportion of rural areas. Lack of community care services and/or access barriers have been linked to fragmented care and a lack of teamwork between stroke survivors, caregivers, and healthcare providers [29]. Service gaps, fragmented care and access issues also shift the burden of care to informal caregivers leading to increased stress/strain and decreased quality of life, which has been highlighted in the literature [19, 30, 31]; with a US study estimating the total national economic burden associated with informal caregiving post-stroke at $14.2 billion [32].

Virtual stroke rehabilitation services (also called telemedicine, telehealth, or telerehabilitation) have been introduced to help address service gaps/access issues. Before the COVID-19 pandemic these services were not widely used; however, the pandemic propelled their rapid expansion in Canada and across the globe to address abrupt interruptions in clinical services [33, 34]. Currently, only a few countries have clinical guidelines regarding stroke telerehabilitation, such as the US [35] and Canada [34]. These guidelines provide a framework for when and how to deliver virtual stroke care and evaluate it; however, they are based on a weak evidence base and very little information on the preferences of people with stroke [33]. Recent systematic reviews cite small effect sizes favouring telehealth for balance and functional mobility/ADLs [36–38], but no difference compared to in-person therapy for outcomes such as depression and quality of life [33]. Two umbrella reviews of telehealth trials included 13 reviews of neurological rehabilitation interventions and concluded that there was some evidence that telehealth rehabilitation may be equivalent or possibly better than in-person services [39, 40]. Importantly, all these reviews note that the evidence to date is based primarily on small, inadequately powered trials with a high risk of Type II error [33, 40]. There is a clear need for stronger studies to provide the necessary evidence to guide the design of effective virtual stroke rehabilitation interventions.

Consideration of telehealth as a replacement for in-person services needs to ensure that certain groups are not disadvantaged. Studies have reported that telehealth services are less likely to be used by certain ethnic groups, rural residents, men, and older adults [41]. Older adults may be particularly challenged by technological solutions and are also vulnerable in other ways. An estimated 50% of all strokes occur in people over 75 years [42–44] and approximately 92% of older adults with stroke have at least 2 other chronic conditions [45]. Multiple chronic conditions have been linked to lack of care coordination/communication in transitions across care providers [19]. Accordingly, older adult stroke survivors tend to be a more medically complex subgroup that face a higher risk of death, worse functional outcomes, and longer hospital stays compared to younger stroke survivors [46]. Consequently, TC services, longer-term support, and the appropriate use of telehealth services are priority issues for older adult stroke survivors, yet few TC interventions have been developed and tested for this vulnerable subgroup. More research is needed on TC interventions aimed at enhancing transitions across care environments for older adults with stroke.

Current Canadian best practice guidelines for managing care transitions following stroke are largely built upon evidence from observational or qualitative studies, or expert consensus [19, 47]. Moreover, most studies providing this evidence have focused on hospital-based initiatives, with few examining the role of outpatient or community-based stroke rehabilitation teams [27]. Where possible and appropriate, evidence from ‘gold standard’ randomized controlled trials should be sought to strengthen the evidence base for community-based stroke rehabilitation interventions targeting older adults with stroke and multimorbidity. The Transitional Care Stroke Intervention (TCSI) trial offers such an opportunity. The TCSI trial was designed to test whether a virtual, 6-month intervention in addition to usual care could improve health outcomes and patient experience and reduce health and social service costs in older adults with stroke and multimorbidity (≥ 2 chronic conditions) who return home, compared with usual care. The feasibility and preliminary effectiveness of the TCSI were previously established in a study conducted with a hospital-based outpatient stroke rehabilitation program [48]. This trial contributes to our understanding of the effectiveness of virtual delivery of stroke rehabilitation services on a range of patient-reported outcome measures (PROMs), for an intervention that aims to improve self-management to enable people with stroke to manage the many care transitions they experience following discharge from the hospital. Moreover, the trial targets older adults with multimorbidity, who are among the most vulnerable and medically complex stroke patient subgroups.

## Methods

### Study design

This study is a multi-site pragmatic Randomized Controlled Trial (RCT) that was conducted in Ontario, Canada (ClinicalTrials.gov: NCT04278794). The study was designed to be highly pragmatic, using the criteria described in the Pragmatic Explanatory Continuum Indicator Summary Version 2 (PRECIS-2) tool [49, 50]. Pragmatic features included: 1) the recruitment of participants representative of the population presenting in the outpatient clinic, 2) the flexible delivery of the intervention by the Interprofessional (IP) Team (Occupational Therapist, Physical Therapist, Registered Nurse, Social Worker, Speech Language Pathologist) from the outpatient rehabilitation programs, 3) the use of patient-relevant outcomes (e.g., quality of life, patient experience), and 4) the use of intention-to-treat analysis. We also note that the trial was initiated 7–8 months after the COVID-19 pandemic started in Canada (March 2020), and triggered several unplanned changes to the trial. *All changes to the trial introduced in response to the extenuating circumstances arising from the pandemic have been described at the end of the Methods section (Modifications Triggered by COVID-19).*

Guidelines used in this study include: the Template for Intervention Description and Replication (TIDier) checklist [51] to structure the description of the intervention, the Consolidated Standards of Reporting Trials (CONSORT) [52] to structure the methods and reporting of the results, and CONSERVE-CONSORT guidelines [53] to guide the reporting of modifications to the study due to COVID-10, as discussed in detail below.

### Participants and recruitment

Participants were recruited from two hospital-based outpatient stroke rehabilitation programs within two geographical areas in Ontario, Canada. Study recruitment was conducted during 2020–2021 and spanned 11 months. Study participants were inpatient older adults 55 years of age or older with a confirmed diagnosis of stroke (first ever or recurrent) and referred to an outpatient stroke rehabilitation program. With verbal consent, older adults meeting these criteria were screened prior to hospital discharge (*n* = 182) by a trained recruiter for potential inclusion and were eligible to participate if they met the following criteria: 1) self-reported at least two chronic co-morbid conditions, 2) planned for discharge from hospital to the community (not long-term care), 3) had access to technology to participate in virtual visits, (e.g., access to a telephone, tablet, or other device with video capabilities, and internet connection), and 4) spoke or understood English or if the individual had limited or no English language proficiency or had an interpreter available.

A trained Research Assistant (RA) contacted potential participants within 24–48 h following discharge from hospital to arrange a telephone interview. The RA administered the Telephone Version of the Montreal Cognitive Assessment (T-MoCA) [54] prior to conducting the baseline interview. Participants were eligible to participate in the baseline interview if they obtained a score of 18 /22 on the T-MoCA [55] or had a substitute decision-maker who could consent on their behalf. The T-MoCA was used as a proxy for assessing capacity. It is a reliable and valid cognitive screen that was selected to detect mild cognitive impairment remotely [55].

### Randomization

Within each study region, participants were assigned to either the intervention or the usual care group following the collection of baseline data, using a centralized web-based software program (RedCap) [56, 57] that ensured concealment of the allocation from the research team and the interventionists. Participants were allocated to the two groups using a 1:1 ratio and in accordance with the randomized sequence of permuted blocks (sizes 2,4,6) determined by the statistician and entered into RedCap.

### TCSI intervention

The 6-month intervention was developed using the Medical Research Council Framework [58] for developing complex interventions. This framework highlights the importance of using theoretical and empirical evidence to inform intervention development. Accordingly, the format and content of the intervention were guided by: 1) the existing evidence on transitional care and stroke interventions [21, 47, 59], 2) theoretical underpinnings of Lorig and Holman’s self-management theory [60], 3) focus groups with older adult stroke survivors, caregivers, healthcare providers, and decision-makers during the pre-trial phase, and 4) findings from a feasibility study with a small group of patients at a hospital-based outpatient stroke rehabilitation clinic [48, 61].

Two study governance structures facilitated the active engagement of patient, providers, and decision-maker stakeholders: Patient Advisory Committee, and the Steering Committee. The Patient Advisory Committee consisted of older adult stroke survivors (excluding trial participants) living in the two study regions. The Steering Committee consisted of decision-makers (e.g., from provincial health branches), representatives from provincial and national stroke networks, patient research partners, directors, and managers from the two study sites, and research team members. The involvement of key stakeholders at each of the study sites and organizations involved in the delivery of community-based stroke rehabilitation was critical to designing the intervention to ensure that all viewpoints were considered.

The description of the intervention follows the TIDieR guidelines [51]. Participants randomized to the TCSI intervention received usual care plus the intervention. The intervention consisted of a 6-month tailored patient-centred intervention delivered by an IP team of healthcare providers from a hospital-based outpatient stroke rehabilitation program. The IP team included an Occupational Therapist, Physical Therapist, Speech Language Pathologist, Social Worker, and a Registered Nurse or Registered Practical Nurse. The Physical Therapist or Occupational Therapist also functioned as a system navigator/care coordinator at the two sites. To avoid contamination with the control arm, all providers that were members of the intervention team did not deliver usual care to participants in the control arm of the study. The Occupational Therapist, Physical Therapist and Speech Language Pathologist at one of the study sites provided both the intervention and usual stroke rehabilitation services to participants in the intervention arm of the study.

To support intervention fidelity, the health care providers received virtual training by the principal investigator and the research coordinator prior to initiation of the intervention to convey key intervention activities, research study procedures, and underlying theories. A standardized training manual was developed from best evidence that included key content pertaining to all aspects of the intervention. Training was adapted to the providers based on a needs assessment completed at each of the sites and included education and role-playing to enhance skills in strengths-based practice. Monthly outreach meetings were conducted to enable the principal investigators, the research coordinator, and the intervention teams to support and monitor intervention implementation and strategize to address any challenges [62].

The TCSI was designed to improve both the quality and experience of hospital-to-home transitions. The core components of the intervention were based on the literature (as described in the introduction) [19], and were designed to support self-management, and included: 1) a post-discharge telephone follow-up call within 2 days of hospital discharge by the Care Coordinator, 2) up to 6 virtual visits delivered by phone or videoconference by a member of the IP team that were an average of one hour in duration (first visit occurred within 2 weeks post-discharge and then monthly thereafter), 3) monthly IP team conferences of the intervention team where the team developed an individualized patient-centred plan of care and engaged in ongoing evaluation of the plan of care at each team conference, 4) ongoing care coordination/system navigation support provided by the Care Coordinator, and 5) an online resource to support self-management and system navigation (My Stroke Recovery Journey website) [63]. The providers had access to a secure tool for communication and information sharing within the team and documenting the care that they provided (SharePoint and Electronic Medical Record). Collaborative goal setting between the IP team, older adults with stroke and their family caregivers fostered active participation of older adults in discussions, planning and shared decision-making regarding their care [19].

The IP team’s main activities during the virtual visits included: 1) conducting a comprehensive assessment of the health and social care needs of older adult participants using standardized tools [19]; 2) use of evidence-based guidelines to prevent and manage stroke [19, 47, 64], multimorbidity [65], and transitions in care [9]; 3) conducting medication review and reconciliation and supporting medication management using best practices [66]; 4) self-management education and support using a strengths-based approach, [19, 67–69]; 5) providing navigational support; 6) discussion and support regarding ongoing use of the My Stroke Recovery Journey Website to support system navigation, and 7) assessing and supporting caregivers. During and between visits, with the client’s consent, the team completed and sent alerts (e.g., medication, depressive symptoms, dementia) to communicate any identified concerns with the primary care physician.

During and between the virtual visits, the care coordinator provided system navigation support that consisted of: 1) identifying and addressing any risk factors for adverse events, e.g., hospital readmissions; 2) arranging community services and follow-up health-care appointments; 3) facilitating communication between the patient, their family caregiver, and their health care team; 4) supporting linkages and referrals to relevant health and social service providers; and 5) developing an individualized patient-centred plan of care [70] in collaboration with the IP team and other health and social care providers in the older adults’ circle of care. Hypothesized mechanisms of action for the TCSI intervention included increased stroke self-management, patient experience, and physical and mental functioning. Consistent with a pragmatic trial design, the intervention was tailored to patient needs and preferences and the local context. For example, patients could decline any number of virtual visits, and all participants continued to have access to the programs and services normally offered in their community.

### Control arm: usual care

Older adults who were randomly assigned to the control group continued to receive usual care services offered through their outpatient stroke rehabilitation program. For the two hospital outpatient clinics, this included the provision of stroke rehabilitation services, appointments to see outpatient medical providers, e.g., primary care, neurology, rehabilitation, and referrals to other community-based health and social services. Usual care typically involved 2–3 visits per week for a maximum of 3 months. The specific services that comprise outpatient stroke rehabilitation differed between the two study sites in terms of access to different types of healthcare providers, proportion of visits offered in person or virtually, and the intensity and duration of rehabilitation. During the study period, because of the pandemic, many in-person outpatient rehabilitation visits were not classified as essential services, resulting in reduced access to in-person care, or closure of the clinic [34]. Up to 70% of outpatient rehabilitation visits were offered virtually with the onset of the pandemic [34]. The details of usual care at each site were carefully documented.

**Fig. 1:**
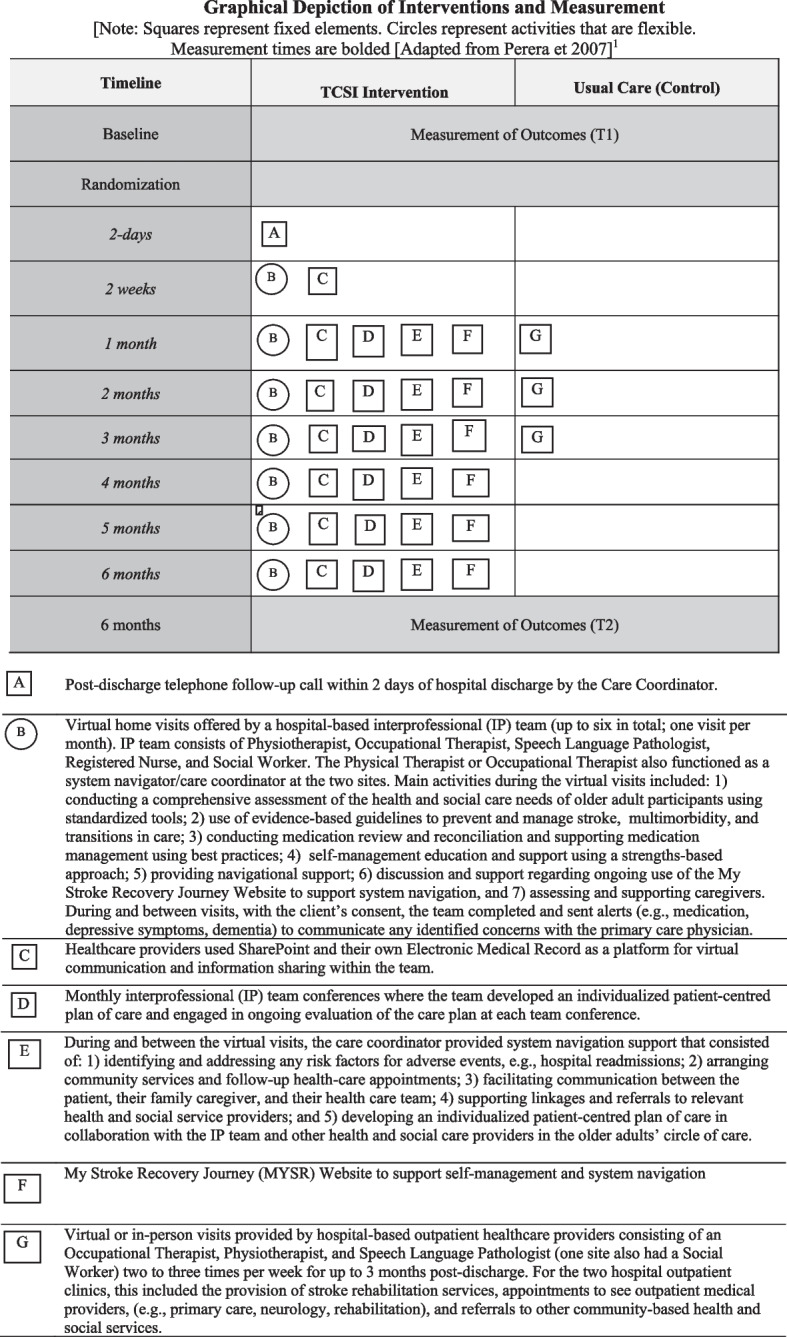
Graphical Depiction of Interventions and Measurement. [Note: Squares represent fixed elements. Circles represent activities that are flexible. Measurement times are bolded [Adapted from Perera et 2007] [71]

Figure 1 provides a summary of the key components of the intervention and elucidates the differences between the intervention and usual care groups. The key differences between the TCSI intervention and usual care (control) that the TCSI introduced included: 1) dedicated Care Coordinator who provided system navigation support and referral to community-based services, 2) regular monthly IP team conferences, 3) regular monthly visits over a 6-month period (usual outpatient rehabilitation services typically last a maximum of 3 months), 4) post-discharge telephone follow-up call within 2 days of hospital discharge by the Care Coordinator. 5) Social Worker and Registered Nurse included in IP team, 6) self-management education and support using a strengths-based approach, 7) use of My Stroke Recovery Journey Website to support self-management and system navigation, 8) standardized clinical assessment tools, including tools to assess for depressive symptoms, and 9) development and ongoing evaluation of an individualized patient-centred plan of care.

### Patient and public involvement

A key component of this patient-oriented research project was the meaningful engagement of diverse patient partners with the lived experience of stroke, in all stages of the research process. The patient partners lived within the study regions and reflected the diversity of older adults with stroke with respect to their sex, living arrangement, marital status, and level of support from a care partner. Patient research partners were actively involved as members on: 1) the Research Steering Committee to provide input on the design of the trial and management oversight, and to inform cross-site implementation of the research; 2) the Patient Advisory Committee, and 3) research team as Co-Investigators. Through these structures, patient research partners assisted with identification of the research priorities and questions, selection of patient-relevant outcomes, refinements to study materials (e.g., consent forms, interview guides), interpretation of study findings, and co-creation of knowledge dissemination products, (e.g., presentations, website, video). Patient engagement was grounded in principles of inclusiveness, support, mutual respect, and co-build [48, 72, 73].

### Outcomes and measures

Outcomes were assessed at baseline and at the 6-month follow-up through interviewer-administered questionnaires during a structured telephone interview. The primary study outcome (risk of all cause hospital readmission) was measured using the Health and Service Utilization Inventory (HSSUI) Browne, Gafni [74], a reliable and valid self-report questionnaire that measures the use of health and social services [75, 76]. Secondary outcome measures aligned with the key components of the intervention, and included: 1) physical and mental functioning measured using the Physical Component Summary Score (PCS) and the Mental Component Summary Score (MCS) from the SF-12 health survey [77], 2) depressive symptoms measured using the Centre for Epidemiological Studies Depression Scale 10-item tool (CES-D-10) [78], 3) stroke self-management measured using the Southampton Stroke Self-Management Questionnaire [79], and 4) patient experience measured using the Person-Centred Coordinated Care Experience Questionnaire (P3CEQ) [80]. These instruments have demonstrated reliability and validity in people with stroke and have been used in our previous trials involving community-living older adults with stroke and multimorbidity [61, 81].

Number of hospital days and readmissions, number of Emergency Department (ED) visits, survival rates to first hospital and ED visit, risk of ED visits, and health and social service use costs were measured using the HSSUI [74, 82]. The HSSUI captures use of primary care, emergency department and specialists, hospital days, other health and social professionals, prescribed medications, and lab services. The cost analysis applied unit costs to the service volumes reported in the HSSUI and assumed a societal perspective to inform the broad allocation of resources in the public interest [83].

### Blinding

To reduce bias, study participants were blinded to their group allocation (usual care, intervention) and the RA’s who collected the assessment data and statistician who analyzed the data were also blinded. Usual care providers were also unaware of the participants’ group allocation. The intervention was known to the providers who were administering the intervention however, they were unaware of the outcomes being studied. Upon completion of the study at 6-months, participants received a debriefing letter describing the two groups and their group allocation.

### Sample size

The target sample size of 96 older adults was based on detecting an effect size of 8% for the proportion hospitalized over the six-month intervention period (primary outcome) as observed in our previous Ontario feasibility study [48]. The calculation assumed 80% power, 2-tailed alpha of 5%, and 20% attrition [48]. A total sample size of 96 required 48 participants per site.

### Statistical analysis

The data are presented as means and standard deviations for continuous variables and numbers and percentages for categorical variables. Analysis of covariance (ANCOVA) was used to test the differences in outcome variables between the intervention and control groups at 6 months. Separate ANCOVA models were run for each outcome, with the 6-month outcome as the dependent variable, group (intervention, control) as the independent variable, and the baseline value of outcomes as the covariate. ANCOVA model assumptions were checked, and robust ANCOVA was run and compared to ANCOVA where serious departures from the parametric model assumptions were observed. Multiple imputation (MI) was used in the primary analysis; it is considered the best method for addressing the most common and realistic missing data patterns seen in RCTs [84]. Joint modelling (JM-MI) was used as it has been found to be like the other most-commonly used MI method (fully conditional specification, FCS-MI) and sometimes outperforms it. Recent evidence has concluded that researchers can use JM-MI with confidence for imputation of missing data for a wide range of data types including continuous, binary, categorical, ordinal and count data [85]**.**

Subgroup analyses were proposed *if* a significant treatment effect was observed for the primary outcome. Four factors were a priori selected for the subgroup analysis: age, sex, number of chronic conditions, and number of prior strokes. Subgroup differences in the intervention effect would be determined based on recommended practice to examine interaction effects in the regression model that includes the main effects and interaction terms. A sensitivity analysis was proposed consisting of only patients with complete outcome data at baseline and 6-months (*n* = 79).

Cost analyses were conducted to compare the costs associated with health service use by participants in the intervention and control groups. The service use that patients reported using the HSSUI at baseline and 6 months after the intervention, was multiplied by the unit costs for the service to obtain total service costs. Unit costs were obtained from public sources (e.g., provincial health agency databases). Cost data are often substantially skewed (as in this study), and non-parametric methods based on ranked data are normally used to analyze group differences. The Mann–Whitney U test was used to evaluate differences in median costs between the two groups [86]. R Version 4.2.2 software was used for all statistical analyses, and a 0.05 (two-tailed) level of significance and 95% confidence was assumed.

### Ethics approval and consent to participate

The study was conducted in accordance with the Tri-Council Policy Statement, Ethical Conduct for Research Involving Humans [87]. Institutional ethics approval was obtained from: the McMaster University Hamilton Integrated Research Ethics Board (REB) (# 8017) and the Hotel Dieu Shaver Health and Rehabilitation Centre REB, and renewed yearly, as required. Operational approval to conduct the study was obtained from each outpatient program site. Written informed consent was obtained from all participants by the RA before study enrolment.

### Modifications triggered by COVID-19 pandemic

In our study, the pandemic resulted in a range of impacts at the sites, described below. As noted in the CONSERVE-CONSORT guidelines, the impact of the pandemic on studies underway “is an exemplar” of extenuating circumstances that are unavoidable and beyond the control of the investigators, funding agencies, and trial sites [53]. These impacts, in turn, triggered important modifications to the study protocol that had potential implications for intervention effectiveness, and ethical acceptability. The research team planned, reviewed, and achieved consensus on the mitigation strategies proposed to address the pandemic impacts. The details of these and other impacts and the mitigation strategies used to address them are outlined below for the CONSORT checklist items that were affected.

#### Intervention

Re-deployment of intervention team members from the outpatient program to acute inpatient units to manage COVID-19 issues resulted in several impacts. Different IP team members (Nurse, Physical Therapist, Occupational Therapist) at one of the sites were re-deployed at various points in time during the intervention period. During those redeployments, participants had limited or no access to these IP team members resulting in different doses of the intervention being offered to participants within and across sites. Re-deployment also impacted attendance at the monthly IP team conferences within sites. The trial had to be stopped 3 months early at one site due to re-deployment of several intervention team members to manage COVID-19 issues. The result was that some participants at this site were offered fewer than 6 visits and discussed less frequently at the IP team conference. It is possible that these impacts could have reduced intervention effectiveness.

Several mitigation strategies were used to address these impacts. The Care Coordinator at each of the sites conducted additional visits to cover for IP team members during redeployments. We tracked the number of visits intervention participants received and number of times they were discussed at the IP team conference to determine if there is a dose–response relationship. The IP team at the study site that stopped the intervention early agreed to offer participants more than one visit per month to make up for the shorter intervention period (e.g., accelerating intervention delivery to increase dose prior to stopping).

#### Harms

The shift in the monthly IP team conferences from the planned in-person to virtual team meetings resulted in the need for additional access to secure virtual technology and the ability to use it effectively to communicate and share information within the team. The teams were concerned that the shift to virtual meetings might impact team collaboration. Several mitigation strategies were used to address these impacts. The IP teams used a web-based collaborative platform (Microsoft Share Point) to securely store study documentation, share them within the team, and keep track of changes or revisions. The Care Coordinator at each of the sites took on the additional responsibility of setting up and maintaining the web-based collaborative platform and trained the members of the intervention team on the use of this software as needed.

Redeployment of members of the intervention team to deal with COVID-19 issues resulted in different doses of the intervention being offered to participants within and across sites and stopping the trial early at one of the sites. These pandemic-related changes could have resulted in a negative impact on the effectiveness of the intervention.

#### Statistical methods

The collective impact of these COVID-related changes on the effectiveness of the intervention is uncertain.

## Results

### Study site characteristics

Table 1 provides information related to the characteristics of the two study sites. Site 1 had a higher proportion of visible minorities and a greater population compared to site 2. In Canada, the visible minority population consists mainly of the following groups: South Asian, Chinese, Black, Filipino, Latin American, Arab, Southeast Asian, West Asian, Korean, and Japanese [88]. Site 2 had a higher proportion of older adults than the provincial average. Site 1 had a higher turnover of intervention team members compared to site 2.

**Table 1:** Study site characteristics

Characteristics	Site 1: Hamilton, Ontario	Site 2: St. Catharines & Niagara Region, Ontario	Ontario
Geographic Density [89, 90]	Urban	Urban/Rural	
Participants Enrolled	45	45	
Intervention Team Turnover	1 RN replaced RPN at 11 months, PT introduced 5 months, and OT replaced 10 months into intervention	None	
Population [91]	~ 569,000	~ 433,600	~ 14,223,942
Languages [91]	92% English 8% Other incl French	91% English 9% Other incl French	92% English 8% Other incl French
Ethnocultural Diversity [91]	26% Visible Minority, 6% FNIM	11% Visible Minority 2% FNIM	22.3% Visible Minority 6.2% FNIM
Proportion of older adults ≥ 55 years [91]	53% (55% Female, 45% Male)	67% (55% Female, 45% Male)	54% (54% Female 46% Male)

*FNIM* First Nations, Inuit, or Metis, *PT* Physiotherapist, *OT* Occupational Therapist, *RPN* Registered Practical Nurse, *RN* Registered Nurse

### Eligibility rate

**Fig. 2:**
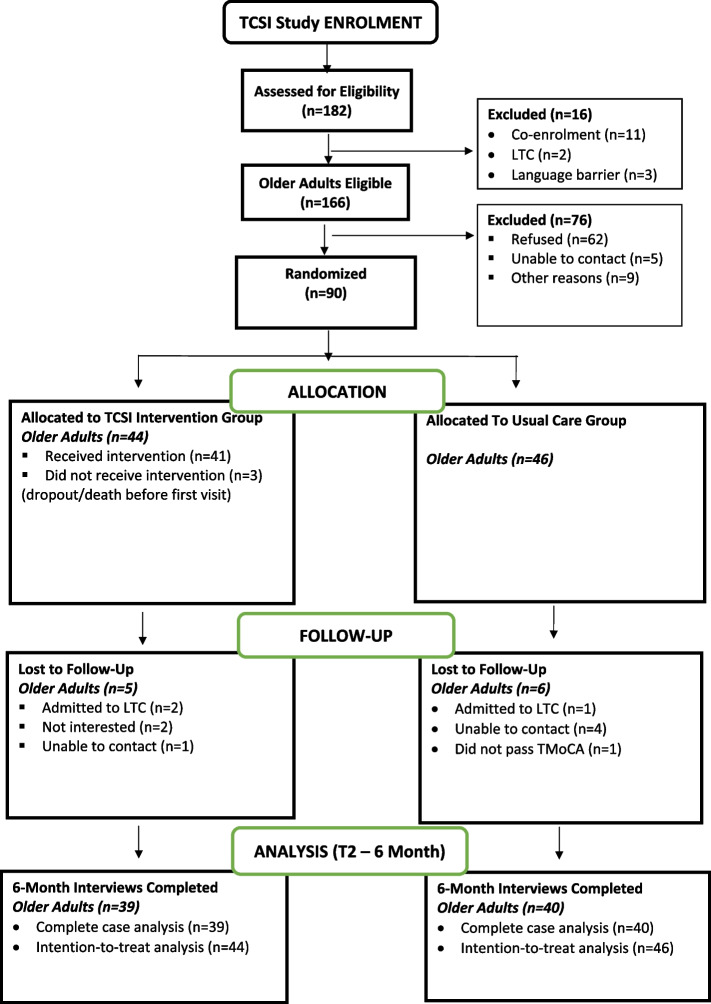
Study flow diagram

Recruitment was conducted over an 11-month period from December 2020 to October 2021. Figure 2 provides a summary of the flow through the trial. A total of 182 consecutive older adults with stroke were screened for the study, and 91% (166/182) met all eligibility criteria. The most common reason for ineligibility (11/16; 69%) was that potential participants were already enrolled in another clinical trial that did not allow co-enrolment.

### Enrolment rate

In total, 54% (90/166) of eligible older adults consented and entered the study. Reasons for refusal to enroll in the study were not interested (40/62, 65%) or feeling overwhelmed (16/62, 26%). Of the 90 enrolled participants, 44 were randomized to the TCSI intervention group, and 46 were randomized to the usual care group.

### Attrition rate

Of the 90 enrolled participants, 79 successfully completed the 6-month follow-up, resulting in a retention rate of 88% (79/90). Reasons for loss to follow-up are shown in Fig. 2. One participant was excluded after randomization because they did not obtain a score of ≥ 18//22 on the T-MoCA and should have been excluded from the start.

### Comparison between dropouts and completers

There were no material baseline differences between the participants who completed the six-month follow-up (*n* = 79) and those who dropped out of the study prior to the six-month follow-up (*n* = 11) on their socio-demographic and health status characteristics.

### Baseline characteristics of participants

Baseline characteristics of the 90 eligible and consenting participants are reported in Table 2. Participants had an average of 7 comorbid chronic conditions and were taking an average of 7 prescription medications daily. Almost all (98%; 89/90) reported a cardiovascular condition in addition to their stroke, 81% (73/90) reported hypertension, 44% (40/90) reported a musculoskeletal condition, 41% (37/90) reported problems with hearing or vision, 40% (36/90) reported diabetes, 29% (26/90) reported a gastrointestinal disorder, and 28% (25/90) reported pain or kidney problems. Most (78%) participants had experienced their first-ever stroke. All participants were within their first six months post-stroke, with an average of 45 days or 6 weeks post-stroke. More than one-quarter (27%) of participants had three or more risk factors for stroke, including physical inactivity (49%), stress (43%), family history of stroke (42%), obesity (36%), poor diet (34%), alcohol use (17%), or smoking (23%).

**Table 2:** Baseline Characteristics of Older Adults with Stroke and Multimorbidity (*n* = 90)

**Characteristics**	**Total (*n*** **= 90)**		**Usual Care Group (*n*** **= 46)**		**Intervention Group (*n*** **= 44)**	
	**n**	**%**	**n**	**%**	**n**	**%**
**Sex**						
Male	54	60.0	29	63.0	25	56.8
Female	36	40.0	17	37.0	19	43.2
**Type of Accommodation**						
House or Apartment	88	97.7	44	95.7	44	100
Retirement Home	2	2.2	2	4.4	0	0.0
**Marital Status**						
Married, living together	59	65.6	27	58.7	32	72.7
Separated, Divorced, Widowed	25	27.7	17	37	8	18.1
Never married	6	6.7	2	4.3	4	9.1
**Education**						
< High School	18	20.0	11	23.9	7	15.9
High School	21	23.3	9	19.6	12	27.2
Post-Secondary	51	56.7	26	56.5	25	56.8
**Employment Status**						
Employed	26	28.9	10	21.8	16	36.4
Unemployed or retired	64	71.1	36	78.2	28	63.6
**Annual Income in CAD**						
> $100,000	14	15.6	7	15.2	7	15.9
$20,000 to $100,000	46	51.1	24	52.2	22	50
< $20,000	10	11.1	4	8.7	6	13.6
**Living Arrangement**						
Live with others	71	78.9	35	76.1	36	81.8
Live Alone	19	21.1	11	23.9	8	18.2
**Access to Informal Support**						
Yes	88	97.8	45	97.8	43	97.7
No	2	2.2	1	2.2	1	2.3
**Ethnicity**						
Caucasian	80	88.9	41	89.1	39	88.6
Other	10	11.1	5	10.9	5	11.4
**Number of Strokes (Lifetime)**						
1	70	77.8	35	76.1	35	79.5
2	14	15.6	9	19.6	5	11.4
> 3 -6	6	6.6	2	4.4	4	9.1
**Number of Risk Factors for Stroke (Range: 0–7)**						
0	11	12.1	6	13.0	5	11.1
1–3	55	61.1	21	67.4	24	54.5
≥ 3	24	26.6	19	19.6	15	34.4
**Type of Risk Factor for Stroke**						
Obesity	32	35.6	14	30.4	18	40.9
Alcohol	15	16.7	6	13.0	9	20.5
Physical Inactivity	44	48.9	20	43.5	24	54.5
Smoking	21	23.3	12	26.1	9	20.5
Stress	39	43.3	18	39.1	21	47.7
Family History of Stroke	38	42.2	19	41.3	19	43.2
Poor Diet	31	34.4	16	34.8	15	34.1
**Type of Chronic Conditions (sample prevalence > 25%)**						
Cardiovascular	89	98.0	45	98.0	43	98.0
Musculoskeletal	40	44.0	23	50.0	17	40.0
Gastrointestinal	26	29.0	12	26.0	14	31.8
Hearing or vision	37	41.1	20	43.5	17	38.6
Diabetes	36	40.0	21	45.7	15	34.1
Pain	25	27.8	14	30.4	11	25.0
Kidney problems	25	27.8	11	23.9	14	31.8
**Depressive Symptoms**						
> 10 (CES-D-10)	22	24.4	12	26.1	10	22.7
< 10 (CES-D-10)	68	75.6	34	73.9	34	77.3
**Number of Prescription Medications**						
0–3 medications	11	12.6	6	13.6	5	11.6
4–7 medications	48	55.2	23	52.3	25	58.1
> 8 medications	28	32.2	15	34.1	13	30.2
	**Total**		**Usual Care Group *n* = 46**		**Intervention Group *N* = 44**	
	**Mean**	**SD**	**Mean**	**SD**	**Mean**	**SD**
Age	69.83	9.41	70.89	9.34	68.73	9.48
Number of Chronic Conditions	7.05	2.79	6.91	2.75	7.20	2.83
Number of Prescription Medications	6.51	2.92	6.50	3.29	6.53	2.69
Depressive Symptoms ^a^	6.33	4.24	6.63	4.17	6.02	4.34
Stroke Self-Management ^b^	115.14	11.82	114.04	12.02	116.42	12.07
Patient Experience ^c^	17.95	4.44	17.74	4.28	18.16	4.57
Physical Functioning ^d^	37.50	8.35	37.15	8.09	37.86	8.70
Mental Functioning ^e^	49.19	8.32	49.29	8.13	49.09	8.61
Number of Days Post-Stroke	44.81	34.29	48.64	41.97	40.81	28.32
Number of hospital admissions, last 6 months	1.19	0.42	1.17	0.44	1.20	0.41
Number of ER visits, last 6 months	1.09	0.59	1.09	0.59	1.09	0.60

^a^Measured by Centre for Epidemiologic Studies Depression 10-item Scale (CES-D-10), scale range 0–30

^b^Measured by Southampton Stroke Self-Management Questionnaire (SSSMQ), scale range 28–168

^c^Measured by the Person-Centred Coordinated Care Experience Questionnaire (P3CEQ), scale range 0- 30

^d^Measured by Physical Component Score (PCS) of the SF-12 Health Survey, scale range 0–100

^e^Measured by the Mental Component Score (MCS) of the SF-12 Health Survey, scale range 0–100

The majority (60%) of participants were men, living with a spouse or other family member (79%), married (66%), and were an average of 70 years of age. Almost one-half (46%) had annual incomes of less than CAD $60,000, 20% had less than a high school education, and 21% lived alone. In 2021, the average annual income in Canada was $55,524 [92]. Almost all (98%) reported receiving some form of support from a family member or friend. One quarter (24.4%) screened positive for depressive symptoms (≥ 10 on CES-D-10).

A higher proportion of participants in the intervention group were married or living together (72.7% vs. 58.7%), employed (36.4% vs. 21.8%), had three or more risk factors for stroke (34.4% vs. 19.6%), including obesity (40.9% vs. 30.4%), and physical inactivity (54.5% vs. 43.5%) compared with the usual care group. A lower proportion of participants in the intervention group reported a musculoskeletal condition (40% vs. 50%) and diabetes (34.1% vs. 45.7%) compared with the usual care group.

### Intervention dose

Of the 44 intervention participants enrolled at baseline, 93% (41/44) received at least one visit by the IP team. Reasons for not receiving the intervention (all or in part) are shown in Fig. 2. Over the 6-month intervention period, these forty-one participants received a median of six virtual visits by one of the IP team providers (offered 6). Three-quarters (76%; 31/41)% received six visits, 20% (8/41) received 4–5 visits, and 5% (2/41) received 2–3 visits.. About one-third of these participants (32%; 13/41) received at least one visit by videoconference; the remainder were conducted by phone. Almost one-half (44%; 92/212) of the total number of visits were provided by the system navigator who also functioned as either a Physical Therapist or Occupational Therapist on the IP team at the two sites. The remainder of the visits were provided by the Occupational Therapist (13%; 27/212), Speech Language Pathologist (13%; 28/212), Social Worker (13%; 27/212), Registered Nurse or Registered Practical Nurse (9%; 20/212), and the Physical Therapist (8%; 18/212). All members of the IP team met monthly over the study period to discuss the study participants. The intervention was delivered as intended with the exception of the COVID-related adaptations. Variation in the number of visits received by participants was appropriate for a tailored, patient-driven intervention.

#### Effects of the intervention

##### Primary outcome

There was no difference between groups in the risk of hospital readmission (all cause) from baseline to six months for the 79 participants who completed the study (RR: 0.62, 95% CI: 0.16, 2.40, *p* = 0.48).

##### Secondary outcomes

**Fig. 3:**
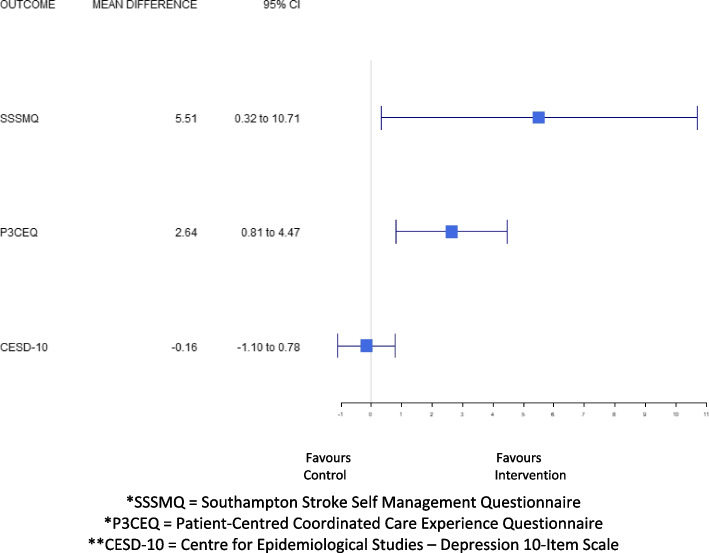
Mean difference in health-related quality of life, stroke self-management, patient experience, and depressive symptoms from baseline to six-months. *SSSMQ = Southampton Stroke Self Management Questionnaire. *P3CEQ = Patient-Centred Coordinated Care Experience Questionnaire. **CESD-10 = Centre for Epidemiological Studies – Depression 10-Item Scale

For the secondary outcomes, the results of the multiple imputation (*n* = 90) showed that there were significant group differences in favor of the intervention on the: SF-12 Physical Component Summary Score (mean difference: 5.10; 95% CI: 1.58–8.62, *p* = 0.005), the Southampton Stroke Self-Management Questionnaire (mean difference: 6.00; 95% CI: 0.51–11.5, *p* = 0.03), and the Person-Centred Coordinated Care Experiences Questionnaire (mean difference: 2.64, 95% CI: 0.81, 4.47, *p* = 0.005). There were no significant group differences between the intervention and control groups on the SF-12 Mental Component Summary Score (mean difference: 2.59; 95% CI: -0.45–5.64, *p* = 0.09), and the CES-D-10 (mean difference: -0.38; 95% CI: -1.31–0.56, *p* = 0.42) (Table 3 and Fig. 3). Complete case analysis results were consistent with the multiple imputation findings.

**Table 3:** Group differences in outcomes from baseline to six-months (*n* = 90)

Outcomes	Group Difference^a^	[95% CI]	*p*-value
	**SF-12 (*n* = 90:** ***n* = 44 Intervention, *n* = 46 Control)**		
Physical function	4.18	[0.31, 8.06]	**0.034**
Role physical	2.66	[-0.73, 6.05]	0.124
Bodily pain	2.93	[0.07, 5.80]	**0.045**
General health	7.28	[3.86, 10.69]	**0.000**
Vitality	5.16	[1.51, 8.81]	**0.006**
Social function	2.48	[-0.79, 5.75]	0.137
Role emotion	1.84	[-1.87, 5.55]	0.330
Mental health	1.73	[-1.45, 4.90]	0.286
Physical Component Summary	4.83	[1.66, 8.01]	**0.003**
Mental Component Summary	2.05	[-0.78, 4.88]	0.155
	**CESD-10 (*n* = 0: *n* = 44 Intervention, *n*- = 46 Control)**		
CESD-10 Score	-0.16	[-1.10, 0.78]	0.734
	**SSSMQ (*n* = 90: *n* = 44 Intervention, *n* = 46 Control)**		
SSSMQ Score	5.51	[0.32, 10.71]	**0.038**
	**P3CEQ (*n* = 90: *n* = 44 Intervention, *n* = 46 Control)**		
P3CEQ Score	2.38	[0.62, 4.14]	**0.008**

^a^Intervention mean – control mean. Result from ANCOVA model adjusted for baseline values. Result generated by pooling ANCOVA model results from individual model runs for 10 imputed data sets

The results of the statistical comparisons for complete cases (*n* = 90) showed that there were no significant differences between the intervention and control groups on the: number of hospital re-admissions (3/39 vs. 5/40; *p* = 0.48), number of hospital days (0.43), survival rate for first hospital re-admission (all cause) (*p* = 0.60), risk of ED visits (all cause) (*p* = 0.14), number of ED visits (1/39 vs. 5/40; *p* = 0.10), or survival rate for first ED visit (all cause) (*p* = 0.10). The median intervention cost was CAD $1,138.00 (interquartile range CAD $1,138.00-$1,158.00) per study participant, there was no statistically significant difference between groups in the change in total costs (including or excluding hospital costs) from baseline to 6-months (*p* = 0.97). For example, cost changes for some services favored the intervention group (other healthcare professionals, home care, supplies and services) and others favoured the control group (physician specialist, prescription medications). However, only two of these differences were statistically significant. There were statistically significant group differences in the change in cost for home care services (*p* = 0.01), and the intervention (*p* < 0.001) favoring the intervention group. (Table 4).

**Table 4:** Group differences in health and social service costs^a^ from baseline to six-months (*n* = 90)

Service^e^	Intervention (*n* = 44)		Usual Care Group (*n* = 46)		Group Differences
	**Baseline** **Median (QI, Q3)**	**6-Month Median (Q1, Q3)**	**Baseline** **Median (QI-Q3)**	**6-Month Median (Q1-Q3)**	**Wilcoxon—W statistic (*p*-value)**^**b**^
Family Physician Visits	84.45 (0.00, 153.22)	247.40 (168.90, 306.20)	126.67 (84,45, 295.53)	232.90 (168.90, 339.2)	970.50 (0.06)
Physician Specialist Visits	69.95 (0.00, 192.78)	118.40 (0.00, 202.40)	77.02 (0.00, 202.37)	162.95 (63.97, 276.04)	665.50 (0.26)
Other Healthcare Professionals	0.00 (0.00, 90.61)	121.20 (0.00, 367.50)	0.00 (0.00, 166.20)	60.00 (0.00, 233.80)	855.00 (0.45)
Home Care	0.00 (0.00, 0.00)	0.00 (0.00, 0.00)	0.00 (0.00, 0.00)	0.00 (0.00, 0.00)	593.00 (0.01)
Prescription Medications	1261.80 (552.80, 2314.90)	1079.00 (440.00 2202.00)	649.70 (295.70, 1664.5)	720.26 (366.98, 1315.39	766.00 (0.89)
Intervention ^c^	0.00 (0.00, 0.00)	1138.70 (1138.70, 1158.20)	0.00 (0.00, 0.00)	0.00 (0.00, 0.00)	1560.00 (<0.0001)
Supplies & Equipment	20.00 (0.00, 104.00)	0.00 (0.00, 0.00)	0.00 (0.00, 75.50)	0.00 (0.00, 0.00)	643.50 (0.15)
Ambulance & 911	249.00 (0.00, 249.00)	0.00 (0.00, 0.00)	249.00 (180.00, 249.00)	0.00 (0.00, 0.00)	840.00 (0.51)
Emergency Department Visits	296.00 (296.00, 296.00)	0.00 (0.00, 0.00)	296.00 (296.00, 296.00)	0.00 (0.00, 0.00)	698.00 (0.32)
Hospital Admissions	31836.00 (15350.00, 49460.00)	3411.00 (2274.00, 5685.00)	31,268.00 (15918.00, 41216.00)	1137.00 (1137.00, 2274.00)	765.00 (0.89)
Total Health and Social Service Costs (including Hospital Costs)	34,623.00 (18163.00, 53934.00)	2918.00 (1971.00, 5504.00)	33682.00 (20044.00, 45093.00)	2157.20 (1057.90, 4368.30)	775.00 (0.97)

^a^Currency CAD

^b^Wilcoxon-Mann–Whitney test is a non-parametric analog to the independent samples t-test. The hypothesis being tested is whether the median differences are equal for the two groups

^c^Includes costs of the intervention (home visits, monthly case conferences, and interventionist training). The mean cost of the intervention per patient was $1,111.70

## Discussion

To our knowledge, the TCSI Study is the first pragmatic RCT designed to enhance the quality and experience of hospital-to-home transitions for older adults with stroke and multimorbidity, 78% of whom had experienced their first-ever stroke, 94% of whom had four or more co-morbid conditions, and 87% of whom were taking four or more prescription medications daily. We found that although the TCSI did not improve hospital readmission rates (primary outcome), it produced greater gains in patient-reported physical functioning, stroke self-management and patient experience compared with usual stroke care. Notably, these improvements were achieved at no additional cost, from a societal perspective. Although we did not directly measure the acceptability of the intervention, the high engagement rate (93%) and low “dropout” rate (11%) over the six-month study period suggests that this approach is highly acceptable to this population.

Our study is innovative for several reasons. First, it involved testing a TC intervention that focused on clinically complex older adults (with stroke and multimorbidity) who are often excluded from studies evaluating TC models [93–96]. Second, it involved measuring the costs of use of a full range of health and social services, from a societal perspective. While there has been some recent work evaluating the cost- consequences of ESD services [97], previous trials assessing the impact of TC interventions provide little information on costs and most have only focused on the cost of institutional care (e.g., hospital, ED visits, long-term care). Costing data will provide policy- and decision-makers with economic information needed to inform decision making related to integrating the intervention into usual care practice. Third, we evaluated the effectiveness of the intervention on *patient-relevant outcomes* within a multimorbidity context (e.g., health-related quality of life, depressive symptoms, patient experience). Previous trials assessing the impact of TC interventions have used limited patient-relevant outcomes (e.g., quality of life, patient experience, stroke self-management) [93]. This is of particular concern as older people with stroke are being discharged early and expected to self-manage their care, and navigate complex and fragmented systems of care independently [93]. Fourth, the intervention was delivered over *6 months* compared with usual care, which is typically delivered over a maximum of 3 months (evidence suggests that community reintegration takes up to one year post-stroke and all participants were within their first 6 months post-stroke) [98]. Fifth, we evaluated the effectiveness of a virtual TC intervention during the COVID-19 pandemic on patient-relevant outcomes. Findings from studies and systematic reviews suggest that virtual stroke rehabilitation services (also called telemedicine, telehealth, or telerehabilitation can be both feasible and effective [99–102]. However, they are based on a weak evidence base, provide very little information on the preferences of people with stroke, and none focus specifically on older adults with stroke and multimorbidity. Moreover, these studies provided little information on the effect of virtual stroke rehabilitation on patient-relevant outcomes. The setting of this study is important because of the increasing emphasis on community-based stroke prevention and rehabilitation services to improve outcomes for older adults with stroke and multimorbidity transitioning from hospital to home and reduce health system costs [27].

Our results are consistent with our previous RCT which studied an IP team approach to community-based stroke rehabilitation for older adults with stroke receiving home care services, that found improvements in physical functioning after 1 year [81]. Our findings are also consistent with those of a systematic review that found that community-based rehabilitation is effective in improving the physical functioning of persons with stroke [3], another Ontario-based study of a community-based stroke rehabilitation team that found significant improvements in the physical domain of the Stroke Impact Scale [103], and a social-worker-led case management program combined with a website providing stroke-related information that found significant improvements in the physical health domain of the Patient-Reported Outcomes Measurement Information System [104].Warner et al. [105] and Sahely et al. [106] found that stroke self-management programs, such as the TCSI, can significantly increase participation, functional ability, and mobility post-stroke. There are four potential reasons for the improvement in physical functioning. First, low-intensity exercises are known to improve oxygen consumption and glucose control – ultimately enhancing physical functioning, 2) physical activity post-stroke help to strengthen muscle performance and lower limb strength – ultimately improving gait speed, 3) connecting participants to health and social services in the community can help them to engage in activities beneficial to improving their physical functioning [3], and 4) providing participants with longer-term rehabilitation and support (6-months versus the usual 3 months of ESD and TC services) [11].

The finding that the TCSI resulted in greater improvements in stroke self-management is consistent with a systematic review that showed that self-management programs improved quality of life and self-efficacy in people with stroke [107]. This finding is expected given that the goal of the intervention was to improve self-management through goal setting, problem-solving, tailored education, self-monitoring and follow-up, and an individualized approach [48]. The finding that the TCSI resulted in greater improvements in patient experience is consistent with the Extended Stroke Rehabilitation service (EXTRAS) trial which found that patients who received virtual assessment by an early supported discharge team member 1- 18 months post-discharge reported greater satisfaction with some aspects of their care than those who received usual care [108]. This finding may also be due to the collaborative, patient-centred focus of the TCSI, that encouraged active participation of older adults in discussions, planning, and shared decision-making regarding their care. Another possible explanation for this finding is that there is evidence to suggest that improvement in patient experience is associated with self-reported improvements in physical functioning [109].

The finding that there were no significant group differences for the primary outcome [baseline to six-month risk of hospital readmission (all cause)] is likely a result of the lack of power (small sample size). The sample size for the trial was based on an effect size of 8% difference in the proportion hospitalized over the six-month intervention period observed in the previous Ontario feasibility study [48]. The difference between the groups may be lower. The effect size and resulting sample size were also based on feasibility considerations, including the total number of potentially eligible participants available, the budget, and the amount of time available. It is possible that the estimated effect size may have been too low to observe an effect with the sample we obtained in the study. Another potential explanation is that during the study period, from 2020 to 2021, hospitals across Canada admitted 11% fewer inpatients, compared with the pre-pandemic period [110]. Nogueira et al. [111] reported an average decline of 19.2% in acute stroke admissions during the height of the pandemic. In a systematic review of 38 studies involving a population who experienced stroke treatment during the COVID-19 pandemic, Van Dusen et al. [112] found that many populations hesitated to seek medical attention, decreasing hospital admissions for less severe strokes by an average of 31%. The conclusion from this review, that included studies from both high- and low-income countries, is that there is clear impact of the COVID-19 pandemic on the admission of patients with a stroke globally. Given that the effect size is the difference in hospital readmissions between the two groups, if overall rates of hospital readmissions were reduced in both groups, the effect size may have been lower than what was originally anticipated, and a larger sample would have been needed to detect the smaller difference.

The older adult participants in the present study are comparable to the general population of community-living adults with stroke described in the literature as reflected by their mean age (71 years) and the proportion of males (60%) [113–116]. However, the average number of self-reported chronic conditions [7] was higher than the average of 5 chronic conditions reported in the literature [117–120]. Caution should be exercised in interpreting this result, however, given the wide variation in the type of chronic conditions measured across the different studies and how they are counted and grouped (e.g., counting heart conditions separately versus including them in one ‘cardiovascular’ grouping). The higher rate of chronic conditions may be due to the nature of our sample. That is, existing studies on stroke rehabilitation often excluded older adults with multimorbidity [121] or a previous stroke, whereas our study included this group. As a result, this study may reflect a group of patients who are more typical of the actual stroke population seen in practice.

Our study has several strengths. A key strength of the trial is that it was highly pragmatic, using the criteria described in the PRECIS-2 tool [50, 122]. Consequently, it reflects the effectiveness of the intervention in real-world clinical practice [123]. Pragmatic features included the recruitment of participants representative of the population presenting in the outpatient stroke rehabilitation program setting, the flexible delivery of the intervention by the IP teams, the use of patient-relevant outcomes (e.g., quality of life, patient experience), the flexible delivery of the intervention by providers, and the use of intention-to-treat analysis [123]. Another strength of the trial was the high rates of enrolment (54%), engagement rates (93%), and follow-up (< 20% dropout). Generally, the enrolment rate observed in this study is high in comparison with other studies of older adult with multimorbidity, such as our previous community-based stroke rehabilitation trial that reported an enrolment rate of 34% [81], the 3D trial that reported an enrolment rate of 33% [124], and the Guided Care cluster trial, where 38% agreed to participate [125]. Attention to intervention fidelity was important for ensuring consistency and adherence to the intervention protocol, which will enhance the potential scalability of the intervention. Several strategies were used to enhance fidelity of intervention implementation, including standardized training of the healthcare providers, regular (monthly) meetings between providers and the research team throughout the intervention period, regular review of documents that provided a record of the intervention components that were delivered, and keeping detailed records of site-specific adaptations made to the intervention. However, the shift from in-person to virtual delivery, and the redeployment of members of the intervention team to deal with COVID-19 issues challenged the fidelity of intervention implementation.

Noteworthy, is that one of the hospital-based outpatient stroke rehabilitation clinic study sites consisted of both urban and rural communities. People who live in rural areas may have difficulties in accessing health care due to a lack of services, isolation, or a lack of mobility [34]. Given the challenge of delivering community-based stroke rehabilitation in rural areas, this virtual intervention may provide an effective and efficient strategy to improve post-acute care for stroke survivors living in rural areas. Prior to the COVID-19 pandemic, virtual stroke care (Telestroke) — which involved connecting with a healthcare provider by email, phone, text, or video call — for acute stroke management were well established in many regions. However, the use of these same technologies to deliver stroke rehabilitation and support reintegration was underused and rare [34].

While many challenges arise conducting pragmatic trials testing complex interventions under ‘normal’ circumstances, managing a trial during a pandemic added an additional layer of complexity, resulting in many uncertainties and unique challenges. A noteworthy strength of the trial was our ability to work together to overcome a broad range of unique methodological and logistical challenges, including consenting and recruiting new participants, data collection and management, and intervention delivery, to name a few. An important strength of the trial was that we were able to revise the intervention from in-person to virtual delivery prior to initiating the intervention, which helped to lessen the impact of COVID on intervention delivery.

### Study limitations

Several limitations to this study should be noted. First, our study took place within two hospital-based outpatient stroke rehabilitation programs in Ontario, Canada, which may limit the generalizability to other settings. Sites may differ on characteristics that can affect implementation of the intervention thereby influencing intervention effectiveness. For example, staffing is known to vary across outpatient stroke rehabilitation programs in Ontario [93]. Future RCTs should involve multiple sites, to explore how the intervention performs across a broader range of settings and contexts. Second, while efforts to identify the key components that make interventions effective have been done in ESD and ASU studies [126, 127], our study was not powered to detect these effects and the complex nature of our intervention suggests that it may be misleading to attribute the effects seen in our study to specific intervention components. Third, the study was conducted during the COVID-19 pandemic. Despite our efforts to minimize the effects of the pandemic, we do not know what would have been the results of the study if the study was conducted under ‘normal’ circumstances, e.g., not under the constraints of the pandemic.

## Conclusions

The results of this pragmatic RCT demonstrated that the TCSI produced greater gains in patient-reported physical functioning, stroke self-management and patient experience for older adults with stroke and multimorbidity compared with usual stroke care. These improvements were achieved without increasing total healthcare costs. Such an approach is highly acceptable to this population and can be implemented in an outpatient stroke rehabilitation setting using existing resources. Improved stroke rehabilitation and recovery during the post-acute period has been identified as a key strategic priority globally to optimize recovery from stroke [128]. This includes designing interventions that directly support stroke survivors and their caregivers during care transitions [5]and address gaps in transitional care. The TCSI has the potential to address this priority by optimizing continuity of care and improving outcomes for older adults with stroke and multimorbidity. The alignment of the project with government policy, the ongoing relationships between researchers and decision-makers, and the use of integrated knowledge translation strategies, will enhance the adoption and implementation of this novel intervention. Future research should involve a larger pragmatic RCT to determine intervention effectiveness in diverse geographic settings and ethno-cultural populations and examine intervention scalability. Future trials should also examine intervention sustainability because many trials, including this trial, measured only short-term effects.

## Data Availability

The datasets used and/or analyzed during the current study are available from the corresponding author on reasonable request.

## Abbreviations

TCSI: Transitional Care Stroke Intervention
IP: Interprofessional
TC: Transitional Care
PRECIS-2: Pragmatic Explanatory Continuum Indicator Summary Version 2
TIDier: Template for Intervention Description and Replication
CONSERVE: CONSORT and SPIRIT Extension for Randomized Controlled Trials Revised in Extenuating Circumstances
CONSORT: Consolidated Standards of Reporting Trials
COVID-19: Coronavirus Disease
T-MoCA: Telephone Version of the Montreal Cognitive Assessment
HSSSUI: Health and Service Utilization Inventory
PCS: Physical Component Summary Score
MCS: Mental Component Summary Score
CES-D: Centre for Epidemiological Studies Depression Scale-10
SSMSQ: Southampton Stroke Self-Management Questionnaire
P3CEQ: Person-Centred Coordinated Care Experience Questionnaire
ED: Emergency Department
MI: Multiple imputation
ANCOVA: Analysis of covariance
J-MI: Joint modelling-multiple imputation
REB: Research Ethics Board
RCT: Randomized Controlled Trial
EXTRAS: Extended Stroke Rehabilitation service
T1: Time 1
T2: Time 2
SF-12: Short Form -12
FNIM: First Nations, Inuit, or Metis
PT: Physical Therapist
OT: Occupational Therapist
RPN: Registered Practical Nurse
RN: Registered Nurse
RA: Research Assistant
